# Seasonal Changes in Mood and Behavior Are Linked to Metabolic Syndrome

**DOI:** 10.1371/journal.pone.0001482

**Published:** 2008-01-23

**Authors:** Reeta Rintamäki, Sharon Grimaldi, Ani Englund, Jari Haukka, Timo Partonen, Antti Reunanen, Arpo Aromaa, Jouko Lönnqvist

**Affiliations:** 1 Department of Mental Health and Alcohol Research, National Public Health Institute, Helsinki, Finland; 2 Department of Health and Functional Capacity, National Public Health Institute, Helsinki, Finland; 3 Department of Psychiatry, Helsinki University Hospital, Helsinki, Finland; James Cook University, Australia

## Abstract

**Background:**

Obesity is a major public health problem worldwide. Metabolic syndrome is a risk factor to the cardiovascular diseases. It has been reported that disruptions of the circadian clockwork are associated with and may predispose to metabolic syndrome.

**Methodology and Principal Findings:**

8028 individuals attended a nationwide health examination survey in Finland. Data were collected with a face-to-face interview at home and during an individual health status examination. The waist circumference, height, weight and blood pressure were measured and samples were taken for laboratory tests. Participants were assessed using the ATP-III criteria for metabolic syndrome and with the Seasonal Pattern Assessment Questionnaire for their seasonal changes in mood and behavior. Seasonal changes in weight in particular were a risk factor of metabolic syndrome, after controlling for a number of known risk and potential confounding factors.

**Conclusions and Significance:**

Metabolic syndrome is associated with high global scores on the seasonal changes in mood and behavior, and with those in weight in particular. Assessment of these changes may serve as a useful indicator of metabolic syndrome, because of easy assessment. Abnormalities in the circadian clockwork which links seasonal fluctuations to metabolic cycles may predispose to seasonal changes in weight and to metabolic syndrome.

## Introduction

Obesity is a big and increasing public health problem worldwide relating to many chronic diseases [1]. In total, 66% of the Finnish men and 49% of women are overweight and have the body-mass index (BMI) of 25 kg/m^2^ or over, and about a fifth of them are classified as severely obese and have the BMI of 30 kg/m^2^ or over. Abnormalities in weight, owing to high caloric intake or low physical exercise for example, may lead to the obesity, hypertension, insulin resistance and abnormal lipid levels. These abnormalities often coincide and have therefore been grouped under the term metabolic syndrome. Metabolic syndrome is a risk factor to coronary artery disease and cardiovascular diseases [2]. Risk factors for metabolic syndrome are abdominal obesity, insulin resistance, elevated blood pressure, high plasma lipid levels. High uric acid levels have also been associated with metabolic syndrome [3].

A range of clinical conditions which are associated with adipose tissue functions display disruptions of the circadian rhythms as well [4]. Moreover, night-shift workers whose rest-activity cycles are reversed tend to develop metabolic syndrome [5]. Furthermore, people who habitually sleep less than 6 or more than 9 hours per night have the increased risk of type 2 diabetes and impaired glucose tolerance [6].

The circadian system regulates many physiological and behavioral rhythms following the 24-hour cycles [7]. A key to adaptation is that it allows an organism to anticipate environmental changes imposed by the rising and setting of the sun, is a self-sustaining oscillator or “clock”. In humans, the principal clock is located in the suprachiasmatic nuclei of the anterior hypothalamus in the brain. Molecular details of the signaling pathways used by the principal clock to reset peripheral clocks are still obscure, but oscillating systemic signals such as hormones, metabolites or core body temperatures appear to play a role. On the other hand, the daily feeding-fasting cycles, physical activity and sleep give feedback to the principal clock.

In the primary feedback loop, the positive elements include transcription factors CLOCK or NPAS2 (alias MOP4) and ARNTL (alias BMAL1 or MOP3) or ARNTL2 (alias BMAL2 or MOP9). These proteins heterodimerize and the subsequent complex initiates transcription of target genes, and another regulatory loop is induced by activating transcription of orphan nuclear receptors which either activate transcription of the Arntl gene or repress the transcription process. One cycle involving both loops takes about 24 hours to be completed and is governed by post-translational modifications and may be modulated by the actions of nuclear receptor co-activators and co-repressors.

A recent study designed to test the relevance of these basic research findings showed in an elegant way that two haplotypes of the ARNTL gene were associated with type 2 diabetes and hypertension [8]. This comparative genetics finding translated from mouse and rat models to human provided the first evidence of a causative role of ARNTL variants in the metabolic syndrome. Deletions of the Clock and Arntl genes are known to end not only with circadian abnormalities but also with metabolic abnormalities of glucose and lipid homeostasis in mice and a phenotype resembling the metabolic syndrome in humans. Mice being homozygous for a loss-of function in the Clock gene have not only abnormal rest-activity cycles but also abnormal patterns of food intake, and these mice eat too much, gain weight and have hyperglycemia and dyslipidemia [9]. Mutations in these genes affect the levels of glucose and triglycerides and contribute to the development of glucose intolerance and insulin resistance in a response to high-fat diet [10]. Interestingly, a targeted disruption of nocturnin encoding a deadenylase in the retina confers resistance to obesity as reflected in lower body weight, smaller visceral fat pads, decreased fat accumulation in the liver, decreased lipogenic gene expression and better insulin sensitivity on the high-fat diet in mice [11].

Nuclear receptors in particular not only take part in the regulation of lipid and carbohydrate metabolism but also are linked to the circadian clockwork [12]. Recently, it was demonstrated that PPARGC1A, a transcriptional co-activator which regulates energy metabolism, stimulates the expression of the Arntl and Nr1d1 genes through co-activation of the nuclear receptors of the NR1F family [13]. Mice lacking PPARGC1A show abnormal diurnal rhythms of activity, body temperature and metabolic rate. The disruption of physiological rhythms in these animals was related to abnormalities in the expression of clock genes and those involved in energy metabolism, suggesting PPARGC1A to be a component which integrates the circadian clock and energy metabolism. Nuclear receptors of the NR4A family, of whom NR4A1 is induced by exposure to light and thereby influenced by the seasonal change in the duration of daylight, are transcriptional regulators of hepatic glucose metabolism [14]. So, in summary, elucidation of the circadian systems biology and the role of hormones in synchronization of the circadian clockwork within and between tissues may bring a new perspective on the relationships between the seasonal phenomena and metabolic disorders [15].

### Study aims

Because the circadian pacemaker of the suprachiasmatic nuclei is a seasonal timepiece as well through its ability to encode the length of day [16], we decided to use the seasonal changes in mood and behavior as a proxy for the circadian clockwork. Since the circadian clockwork is linked to energy metabolism and its abnormalities indicate metabolic syndrome in animal models, the seasonal changes as a phenotype have relevance to metabolic syndrome in humans as well. Our aim herein is to analyze the association of seasonal changes with metabolic syndrome at the population level. On the basis of animal models, we hypothesize herein that persons with metabolic syndrome have more seasonal changes in their mood and behavior.

## Methods

The Health 2000 Study was a nationwide health examination survey. The study was carried out in Finland, a north-eastern (60–70°N, 20–31°E) European country with about 5 million inhabitants. The fieldwork with data collection was carried out between September 2000 and July 2001. The two-stage stratified cluster sampling design was planned by Statistics Finland. The sampling frame comprised adults aged 30 years and over living in mainland Finland. This frame was regionally stratified according to the five university hospital regions, each containing roughly one million inhabitants. From each university hospital region or catchment area, 16 health care districts were sampled as clusters (80 health care districts in the whole country, including 160 municipalities). The 15 biggest health care districts in the country were all selected in the sample and their sample sizes were proportional to population size. The remaining 65 health care districts were selected by systematic probability proportional to size sampling in each stratum, and their sample sizes (ranging from 50 to 100) were equal within each university hospital region, the total number of persons drawn from a university hospital region being proportional to the corresponding population size. The 80 health care districts were the primary sampling units, and the ultimate sampling units were persons who were selected by systematic sampling from the health centre districts. From these 80 health care districts, a random sample of individuals was drawn using the data provided by Population Register Centre. Its population information system contains the official information for the whole country on the Finnish citizens and aliens residing permanently in Finland.

For this study, all the persons aged 30 or over (n = 8028) identified and selected by The Social Insurance Institution of Finland were contacted. Interviewers attended training sessions on the specific themes that were to be covered in the computer assisted interviews. During the interviews, the respondents were handed an information leaflet, an informed consent form for signing, and a questionnaire that interviewees were asked to fill in and bring along to the health status examination. All examinees had been asked to come to the health status examination fasting and without drinking on the same day. In the laboratory, a nurse recorded how these instructions had been followed and then took the blood samples. The samples were centrifuged at the examination site and placed into deep freezers at −20°C before they were transferred within one week to the National Public Health Institute and stored in deep freezers at −70°C.

Of the final sample of 7979 persons, 6986 (88%) were interviewed at home or institution face to face and 6354 (80%) attended the health status examination in a local health center or equal setting, while 416 took part in the health status examination at home or in an institution. Overall, 84% participated either in the health status examination proper or in the examination at home [17].

### Assessment of metabolic syndrome

Metabolic syndrome was assessed using the US Adult Treatment Panel III of the National Cholesterol Education Program (NCEP-ATPIII) criteria [18] and defined as having at least three of the following components: the fasting blood glucose level was 6.1 or higher, the blood pressure was high (systolic pressure was 130 mmHg or more or diastolic pressure was 85 mmHg or more), the serum triglycerides level was 1.7 or higher, the serum high-density lipoprotein cholesterol level was lower than 1.0 for men or lower than 1.3 for women, or the waist circumference was 102.1 cm or more for men or 88.1 cm or more for women.

### Assessment of seasonal changes and depressive symptoms

The items of seasonal variation in mood and behavior were taken and adapted from the Seasonal Pattern Assessment Questionnaire [19]. Two modifications were made to the original scoring as follows. Each item was scored from 0 to 3 (none, slight, moderate or marked change), not from 0 to 4 (none, slight, moderate, marked or extremely marked change), with the sum or global seasonality score (GSS) ranging from 0 to 18. In addition, the Seasonal Pattern Assessment Questionnaire (SPAQ) has a question: “If you experience changes with the seasons, do you feel that these are a problem for you?”. This item was scored from 0 to 4 (none, mild, moderate, marked or severe problem), not from 0 to 5 (none, mild, moderate, marked, severe or disabling problem). The questionnaire was translated into Finnish and then back-translated in order to revise the linguistic accuracy.

Since the seasonal changes in mood and behavior were assessed with a modified questionnaire, we tested its psychometric properties. The factor matrix was computed according to the maximum likelihood principle and employing the algorithm presented by Jöreskog [20], and the standard orthogonal Varimax rotation was used to examine the degree of correlation among factors. The reliability estimates were computed according to the principles given by Cronbach [21] and the general measurement framework introduced by Tarkkonen [22] and developed further by Vehkalahti [23] using the RELIAB module of the Survo MM program (www.survo.fi/mm/english.html). The modified questionnaire was thereafter applied for the case assessment using criteria similar to the original case-finding definition for seasonal affective disorder [24]. Each of the six items were taken as a trait and modeled as the dependent variable, as genetic effects appear to have a global influence across all these changes [25]. A two-factor solution was found to describe best the pattern of covariation of these six items, so our analysis disagreed with the assumption of a single global factor. The calculation of reliabilities yielded Cronbach's alpha of 0.81 and the all-item unweighted sum of 0.85 according to a general model with the assumption that measurement errors may correlate. The factor one (weight, appetite) explained 53%, whereas the factor two (sleep length, social activity, mood, energy level) explained 89% of the variance in the global score.

The behavioral manifestation and symptom intensity of depression were assessed as self-report using a modification of the 21-item Beck Depression Inventory [26] as adapted and validated for the Finnish population (www.kela.fi/in/internet/liite.nsf/NET/110607141642EK/File/tutkimuksia86.pdfOpenElement), with the sum score or Beck Depression Index (BDI) ranging from 0 to 55.

As part of the assessment, the participants filled in items concerning leisure time exercise and alcohol use during the past 12 months. The intensity of physical exercise was categorized as follows: low (no strenuous exercise such as reading, watching television or handicraft), medium (lightly strenuous exercise such as walking or bicycling for four or more times a week), keep-fit (fitness training for three or more hours a week), and sport (sports for several times a week). The frequency of alcohol use was categorized as follows: none, low (once to six times a year), medium (once to four times a month), and high (twice to seven times a week).

### Laboratory tests

Routine laboratory tests included the concentrations of blood glucose and those of serum total cholesterol and triglycerides (Glucose Hexokinase, Cholesterol CHOD PAP and Triglycerides GPO PAP, Olympus System Reagent, Germany), those of high-density lipoprotein (HDL) cholesterol and low-density lipoprotein (LDL) cholesterol (HDL-C Plus and LDL-C Plus, Roche Diagnostics GmbH, Germany), and those of gamma-glutamyltransferase (GGT) and uric acid (IFCC/ECCLS and URIKAASI PAP, Konelab, Thermo Electron Oy, Finland). The waist circumference (in centimeters) was measured on the naked waist half way between the iliac crest and the lowest rib at the end of light expiration while the examinee was standing. The height (in centimeters) and weight (in kilograms) were also measured, and the body-mass index (BMI) was calculated.

### Ethics

The study project was coordinated by the National Public Health Institute and implemented in collaboration with the Ministry of Social Affairs and Health. Written informed consent was given by each participant, and before the signing, the protocol had been fully described to all. The procedures followed were in accordance with the ethical standards of the responsible committee on human experimentation and with the Declaration of Helsinki, its amendments and revision.

### Statistics

The data were weighted to take into account the sampling design and to reduce the bias due to non-response. The R project for Statistical Computing (R, version 2.2.1) was applied for, and its survey Package, available through the Comprehensive R Archive Network family of internet sites (www.r-project.org), was run for the analysis of data using survey-weighted generalized linear models for logistic regression analysis. Metabolic syndrome in two categories (no or yes) was included as the dependent variable in all the models.

First, the univariate models were calculated for the following explanatory variables: the sex, age in four categories (30 to 45, 46 to 60, 61 to 75 or 76 to 99 years), area of living in two categories (the southern or northern part of Finland), education in three categories (low, middle or high), marital status in two categories (living alone or with someone), physical exercise, alcohol use, and the BDI in three categories (0 to 9, 10 to 18 or 19 to 55), the GSS in two categories (0 to 7 or 8 to 18), and the six items of which the GSS is comprised in two categories each (no or yes).

Finally, the multivariate models were formulated. For the first multivariate model the sex, age, area of living, education, marital status, physical exercise, alcohol use, and the BDI in addition to the GSS were entered as independent explanatory variables, whereas for the second multivariate model the GSS was replaced by the six items of which the GSS is comprised. In addition, post hoc criterion-specific associations of metabolic syndrome to the items of seasonal changes in mood and behavior were calculated.

## Results

Of the 8028 study participants (4391 women and 3637 men), the assessments of the presence of metabolic syndrome and of the seasonal changes in mood and behavior was a complete one for 5981 individuals who were subsequently included in the risk analysis. Concerning the general population of Finland, the estimated number of individuals with metabolic syndrome together with a global seasonality score equal to winter blues or more severe seasonal changes in their mood and behavior was 232,577 persons aged 30 or over (Table 1).

**Table 1:** Number of individuals w/o metabolic syndrome among the participants and their number being representative of the Finnish population aged over 30 years.

	Study participants		Nationwide population	
	Metabolic syndrome	Metabolic syndrome	Metabolic syndrome	Metabolic syndrome
	No	Yes	No	Yes
GSS				
0	290	135	157,806	73,694
1	236	109	128,519	59,117
2	377	151	205,076	82,037
3	482	170	262,428	92,268
4	569	209	309,467	113,674
5	490	197	266,419	107,398
6	622	244	338,539	133,030
7	337	160	183,096	87,089
8	266	152	144,515	82,916
9	194	106	105,309	57,866
10	134	60	72,940	32,519
11	72	40	39,376	21,701
12	58	38	31,660	20,899
13	25	16	13,420	8725
14	14	8	7354	4559
15	8	3	4535	1701
16	2	1	1058	517
17	2	0	1176	0
18	2	2	1103	1174
GSS				
0 to 7	3403	1375	1,851,349	748,308
8 to 18	776	427	422,447	232,577

As supposed because of the nature of metabolic syndrome and its diagnosis, individuals with metabolic syndrome had not only bigger BMI (t = −42.7, P<0.001) and waist circumference (t = −47.2, P<0,001) but also higher levels of glucose (t = −19.2, P<0.001), total cholesterol (t = −13.2, P<0.001), HDL cholesterol (t = 48.5, P<0.001), LDL cholesterol (t = −11.9, P<0.001) and triglycerides (t = −37.5, P<0.001) than the remaining (Table 2). Levels of GGT (t = −10.2, P<0.001), despite reporting of less alcohol consumption, and those of uric acid (t = −26.6, P<0.001) were also higher in individuals with metabolic syndrome than in the remaining.

**Table 2:** Key characteristics of individuals w/o metabolic syndrome.

	No metabolic syndrome			Metabolic syndrome		
	Mean	95% CI	SD	Mean	95% CI	SD
Age, years	50.8	50.3 to 51.2	14.8	59.8	59.1 to 60.4	15.3
Glucose, mmol/l	5.3	5.3 to 5.3	0.7	6.1	6.0 to 6.2	1.8
Cholesterol, mmol/l	5.8	5.8 to 5.8	1.1	6.2	6.2 to 6.3	1.2
HDL cholesterol, mmol/l	1.5	1.4 to 1.5	0.4	1.1	1.1 to 1.1	0.3
LDL cholesterol, mmol/l	3.6	3.6 to 3.6	1.0	4.0	3.9 to 4.0	1.1
Triglycerides, mmol/l	1.2	1.2 to 1.3	0.6	2.4	2.3 to 2.5	1.3
GGT, U/l	31.8	30.6 to 33.0	38.9	46.0	43.4 to 48.6	56.7
Uric acid, µmol/l	283.6	281.3 to 285.8	73.8	340.5	336.7 to 344.4	83.3
Body-mass index, kg/m^2^	25.4	25.3 to 25.5	3.8	30.3	30.1 to 30.5	4.4
Waist circumference, cm	88.2	87.8 to 88.5	11.4	102.7	102.2 to 103.2	11.5

### Univariate risk analysis

Seasonal changes in mood and behavior were associated significantly with metabolic syndrome (Table 3). Of the explanatory variables, older age, greater depressive symptoms (BDI), greater seasonal symptoms (GSS), and the northern area of living were significant risk factors of metabolic syndrome. The protective factors of metabolic syndrome included greater physical exercise, higher education, living together with someone, and greater alcohol use. Of the six items of which the GSS is comprised, the seasonal changes in weight and appetite were significant risk factors, whereas the seasonal change in social activity was a significant protective factor of metabolic syndrome.

**Table 3:** Protective and risk factors of metabolic syndrome in the Finnish population aged over 30 years (the univariate models).

Explanatory variable	Estimate	Standard error	Odds ratio	95% confidence interval
Sex				
male	reference			
female	0.06	0.05	1.07	0.96 to 1.18
Age				
30 to 45	reference			
46 to 60	0.81	0.07	2.24	1.94 to 2.59
61 to 75	1.26	0.08	3.51	2.98 to 4.14
76 to 99	1.61	0.10	5.03	4.12 to 6.13
Area of living				
southern	reference			
northern	0.25	0.08	1.28	1.09 to 1.51
Education				
low	reference			
medium	−0.66	0.06	0.52	0.46 to 0.59
high	−1.00	0.07	0.37	0.32 to 0.43
Marital status				
alone	reference			
with someone	−0.24	0.06	0.79	0.70 to 0.88
Exercise				
low	reference			
medium	−0.48	0.05	0.62	0.56 to 0.69
fitness	−0.84	0.09	0.43	0.36 to 0.51
sports	−1.27	0.29	0.28	0.16 to 0.49
Alcohol use				
low	reference			
medium	−0.42	0.07	0.66	0.58 to 0.76
high	−0.57	0.09	0.57	0.47 to 0.68
none	0.44	0.08	1.55	1.32 to 1.83
BDI				
0 to 9	reference			
10 to 18	0.28	0.07	1.33	1.16 to 1.52
19 to 55	0.47	0.11	1.60	1.30 to 1.96
GSS				
0 to 7	reference			
8 to 18	0.31	0.07	1.36	1.18 to 1.57
Seasonal change				
no	reference			
yes: sleep length	−0.04	0.06	0.96	0.86 to 1.09
yes: social activity	−0.18	0.06	0.84	0.74 to 0.95
yes: mood	−0.04	0.06	0.96	0.85 to 1.08
yes: weight	0.38	0.05	1.46	1.32 to 1.61
yes: appetite	0.24	0.06	1.27	1.14 to 1.42
yes: energy level	−0.01	0.06	0.99	0.87 to 1.12

### Multivariate risk analysis

First, individuals with the greater global score on seasonal changes in mood and behavior had a significantly higher risk for metabolic syndrome (Table 4). The magnitude of their effect as a risk factor was equal to that of fitness training as a protective factor. Older age and the northern area of living were significant risk factors, whereas greater physical exercise, greater alcohol use, higher education and the female sex were significant protective factors of metabolic syndrome.

**Table 4:** Protective and risk factors of metabolic syndrome in the Finnish population aged over 30 years (the first multivariate model).

Explanatory variable	Estimate	Standard error	Odds ratio	95% confidence interval
Sex				
male	reference			
female	−0.22	0.06	0.80	0.71 to 0.91
Age				
30 to 45	reference			
46 to 60	0.71	0.08	2.03	1.75 to 2.35
61 to 75	1.06	0.09	2.89	2.40 to 3.47
76 to 99	1.26	0.14	3.52	2.70 to 4.59
Area of living				
southern	reference			
northern	0.19	0.09	1.21	1.01 to 1.46
Education				
low	reference			
medium	−0.22	0.08	0.81	0.69 to 0.94
high	−0.42	0.09	0.66	0.55 to 0.79
Marital status				
alone	reference			
with someone	0.08	0.07	1.08	0.95 to 1.24
Exercise				
low	reference			
medium	−0.45	0.06	0.64	0.57 to 0.73
fitness	−0.68	0.11	0.50	0.41 to 0.63
sports	−1.02	0.32	0.36	0.19 to 0.68
Alcohol use				
low	reference			
medium	−0.30	0.08	0.74	0.63 to 0.87
high	−0.43	0.10	0.65	0.53 to 0.80
none	0.15	0.11	1.17	0.94 to 1.45
BDI				
0 to 9	reference			
10 to 18	−0.10	0.08	0.91	0.78 to 1.05
19 to 55	−0.01	0.13	0.99	0.77 to 1.27
GSS				
0 to 7	reference			
8 to 18	0.44	0.08	1.56	1.32 to 1.84

The risk of metabolic syndrome due to the seasonal changes remained similar, that is a significant one, whether they were analyzed as a continuous or categorized global score. The GSS as a continuous explanatory variable yielded the odds ratio of 1.06 (95% CI = 1.04 to 1.08) for metabolic syndrome.

All the explanatory variables were of significance as analyzed separately, with the exception of the sex. However, the sex became to be of significance in the multivariate models. Of the non-seasonal explanatory variables, the same ones remained to be of significance in a similar way in the second multivariate model than in the first one, except the area of living which lost the significance. Of the six items of which the GSS is comprised, only the seasonal change in weight had a significant effect, being a risk factor (Table 5).

**Table 5:** Protective and risk factors of metabolic syndrome in the Finnish population aged over 30 years (the second multivariate model).

Explanatory variable	Estimate	Standard error	Odds ratio	95% confidence interval
Sex				
male	reference			
female	−0.24	0.06	0.79	0.69 to 0.89
Age				
30 to 45	reference			
46 to 60	0.71	0.08	2.03	1.75 to 2.36
61 to 75	1.04	0.10	2.82	2.34 to 3.40
76 to 99	1.29	0.14	3.64	2.78 to 4.77
Area of living				
southern	reference			
northern	0.18	0.10	1.19	0.99 to 1.44
Education				
low	reference			
medium	−0.20	0.08	0.82	0.70 to 0.96
high	−0.40	0.09	0.67	0.56 to 0.80
Marital status				
alone	reference			
with someone	0.07	0.07	1.08	0.94 to 1.23
Exercise				
low	reference			
medium	−0.45	0.07	0.64	0.56 to 0.72
fitness	−0.68	0.11	0.50	0.41 to 0.63
sports	−1.05	0.33	0.35	0.18 to 0.67
Alcohol use				
low	reference			
medium	−0.31	0.08	0.73	0.63 to 0.86
high	−0.42	0.11	0.66	0.53 to 0.81
none	0.16	0.11	1.18	0.95 to 1.46
BDI				
0 to 9	reference			
10 to 18	−0.06	0.08	0.94	0.81 to 1.10
19 to 55	0.07	0.12	1.07	0.85 to 1.36
Seasonal change				
no	reference			
yes: sleep length	−0.00	0.08	1.00	0.85 to 1.17
yes: social activity	−0.10	0.09	0.91	0.76 to 1.09
yes: mood	0.01	0.09	1.01	0.85 to 1.20
yes: weight	0.45	0.06	1.57	1.39 to 1.78
yes: appetite	0.12	0.08	1.13	0.97 to 1.32
yes: energy level	−0.02	0.09	0.98	0.82 to 1.18

### Post hoc analysis

Of the assessment criteria for metabolic syndrome, after controlling for the sex and age of the participants, the waist circumference was correlated significantly to each seasonal change item, but most strongly to the seasonal change in weight (r = 0.20, P<0.001) followed by the seasonal changes in appetite (r = 0.12, P<0.001). All the assessment criteria were significantly associated with the seasonal change in weight.

## Discussion

Our results herein show that there is a significant association of the seasonal changes in mood and behavior with metabolic syndrome. Individuals with metabolic syndrome had greater seasonal changes in mood and behavior. Each point scored on the sum increased the risk of metabolic syndrome by 6%, indicating that the maximum score would end in the 2.1-fold increase in the risk. The risk of having metabolic syndrome was heightened by 56% among those having a global score equal to winter blues or more severe seasonal changes in their mood and behavior. The negative effect of these changes was equal to the positive effect of physical exercise of fitness training to sports activities. Of the assessment criteria for metabolic syndrome, the waist circumference was significantly associated with each seasonal change item and all criterion items were significantly associated with the seasonal change in weight.

We also found that elevated uric acid levels were associated with metabolic syndrome in both genders. This finding agrees with previous studies [3], [27]–[29] and fits in that type 2 diabetes increases the risk for uric acid stones through increasing insulin resistance [30]. Recently, it was demonstrated that higher serum levels of uric acid were associated with a decreased risk of Parkinson's disease [31]. Low levels of uric acid in the substantia niagra contribute to [32] whereas uric acid seems to prevent [33] cell death in animal models of Parkinson's disease.

Earlier, in a study of a convenience sample, the 195 adult persons had a net weight gain of 0.48 kg on average (standard deviation of 2.22 kg) in late February or March as compared with their weight in late September or early October [34]. Since the weight gain was not reversed during the spring or summer months in those 165 participants who returned for follow-up the next September or early October, the net weight gain during the fall and winter is likely to contribute to the increase in weight which frequently occurs during adulthood. Our findings herein suggest that greater seasonal changes in weight, whether they are associated with such net weight gain or not which is likely to predispose to overweight, are linked to metabolic syndrome among individuals aged 30 or over.

How the circadian clockwork or seasonal variation may be linked to energy metabolism? There are at least two possibilities. First, it may be that the circadian clock has evolved to enable metabolic processes to occur harmoniously within a cell, and thereby the circadian cycle is fundamentally a metabolic cycle [35]. The circadian, sleep-wake and seasonal cycles may each be regarded to reflect an intrinsic metabolic cycle. In other words, these metabolic cycles are fuel for and form the input to the circadian clockwork. This point of view extends the two-process model of sleep and its amendment [36] into a new model in which the sleep onset is a key to the timepiece and a switch for the metabolic and cell division cycles from daytime to night-time settings through the actions of the proteins nocturnin [15] as for example. It seems that the metabolic, cell division and circadian cycles may be coordinated similarly as a evolutionarily conserved means of preserving genome integrity [37].

Second, exposures to light, or the light-dark transitions, are needed for reset of the principal circadian clock on a daily basis. When these signals are missed, the circadian clockwork relies more on the metabolic cycles producing time-giving signals needed for adaptation [38]. During hibernation for example the principal circadian clock stops acting as a timepiece [39] but the metabolic futile cycle burning up energy in peripheral organs provides a necessary circadian signal [40]. Changes of season challenge a switch between the metabolic and circadian based time-keeping mechanisms of action. Spring and fall are the periods which challenge the circadian clockwork and its integration. In spring, the morning-tagged cells yield the dominance to the evening-tagged cells [41], or from the wake-up to sleep onset process, within the principal circadian clock. This may need the input from the circannual pacemaker which reacts to changes of season, or changes in the length of day, and drives its targets [42]. In particular, the integration of signals from light exposures and ambient temperatures may be of key importance to the regulation of the circadian clockwork [43]. In spring, oscillations in ambient temperature are robust and often span the lower boundary of the physiological range for rhythm generation, and individuals with abnormalities in the circadian clockwork may have dramatically compromised temperature sensitivity and the subsequent behavioral phase shifting properties [44]. In such cases, the nuclear receptor coactivator 3 which integrates these signals [45] and regulates the white adipogenic program [46] may substitute the compromised functions according to a co-activator hypothesis [47]. It or another co-activator may keep the circadian clockwork in synchronization in conditions similar to Clock mutant [48] or Crem mutant [49]–[51] mice.

Light exposure to the eye stimulates the use of dopamine in the retina following a dose-response relationship [52]. Dopamine is needed for melanopsin expression for instance [53] which takes place under control of a circadian clock [54]. Scheduled exposures to visible light using for example systems biology based [55] or molecular timetable [56] methods in the future may be applied for the treatment of a range of medical conditions to whose pathogenesis the circadian clock genes contribute such as mood disorders with the seasonal pattern [57], [58]. We show herein that the seasonal changes in weight are a risk of metabolic syndrome. This risk may be reduced with the use of not only physical exercise but also scheduled light exposures at appropriate times of the day in order to reset the circadian clock and normalize the output of the circadian clockwork as seen in patients with seasonal affective disorder [59] or bulimia [60]. Such methods may also be of use in the prevention of death from suicide, since the conflicting signals of light-dark cycles in relation to temperature may predispose individuals suffering from a depressive episode to self-threatening behavior on still cold days during spring [61].

Our findings were consistent concerning the effects of the seasonal changes in mood and behavior, physical exercise and alcohol use on the risk of metabolic syndrome. As they all affect the circadian clockwork, our results herein give support to the hypothesized links between the metabolic and circadian cycles generated and guided by the circadian pacemaker [35]. Moreover, our results of a general population now extend these links to include relationships between the metabolic and seasonal fluctuations as well. Reciprocal regulatory loops thereby consist of the metabolic, cell-division, circadian and seasonal oscillations.

### Strengths and limitations of the study

Our findings are representative of the general population aged over 30 living in Finland, a northern European country, and can therefore be generalized directly to concern any population with a similar standard of living at the time of the study.

A limitation is that we assessed the seasonal changes in mood and behavior using a self-report questionnaire. However, the questionnaire is retrospective to the routine seasonal changes during lifetime and has excellent sensitivity and specificity (94% and 73% respectively) for seasonal affective disorder and its subsyndromal form [62], a high internal consistency [63], and two-month test-ratest reliability [64]. Our analysis herein not only confirms its good psychometric properties but also indicates that the global seasonality score is in fact composed of two separate factors having relevance to mood and energy metabolism respectively. The latter factor was associated in specific with and thereby might be a good proxy for metabolic syndrome. However, the questionnaire was not a good case-finding instrument for mental disorders, which agrees with earlier reports [65].

### Conclusion

At population level individuals with metabolic syndrome had more seasonal changes in their mood and behavior, those in weight in particular being a significant risk of having metabolic syndrome. Circadian abnormalities and seasonal changes in weight need to be part of the assessment in persons being at risk of or having metabolic syndrome. If there were seasonal changes in weight, treatment options including scheduled exposures to light might be considered.
